# Supplementary material for: Infection length and host environment influence on Plasmodium falciparum dry season reservoir
Source: EMBO Mol Med. 2024 Sep 16;16(10):2349–75. doi: 10.1038/s44321-024-00127-w (PMC11473648; doi:10.1038/s44321-024-00127-w)
Supplement: Supplementary file 1 — Appendix [file 44321_2024_127_MOESM1_ESM.pdf]

Appendix

Table of contents

Appendix figure S1 -- page 2
Appendix figure S2 -- page 3
Appendix figure S3 -- page 4
Appendix figure S4 -- page 5

Appendix figure S1

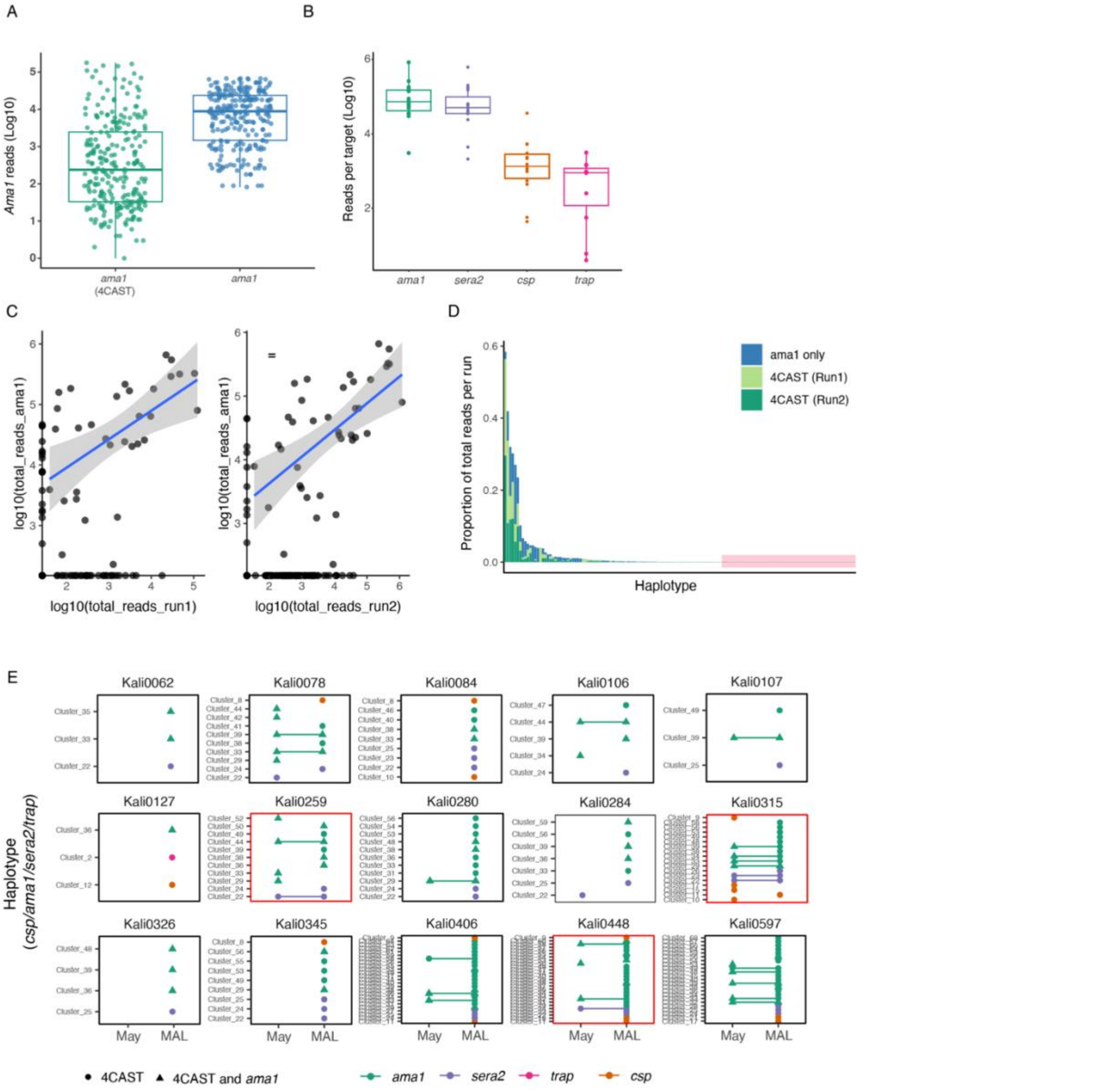

A. Log10 reads of *ama1* detected through *ama1* single amplicon sequencing (n=15) or through multiplexed amplicon sequencing (4CAST, n=15). **B.** Log10 reads of each amplicon target detected by multiplexed amplicon sequencing (4CAST). **C.** Pearson correlation of the total reads (log10-transformed) between two 4CAST runs (x-axis) and ama1 amplicon sequencing (y-axis) for paired individuals (n=15), (R=0.52, p-value = 0.0021 for run 1 and 0.56, p-value = 0.00021 for run 2). Shaded curve indicates the linear regression model with 95% confidence interval. **D.** Distribution of haplotype frequencies (proportion of haplotype reads over total reads in run) for the two 4CAST runs (light and dark green for run 1 and run 2, respectively) and ama1.Haplotypes are ordered by decreasing frequencies and haplotypes detected only in one of the two 4CAST runs are marked with a red box. **E.** Haplotypes of 4CAST targets (*ama1, csp, sera2 or trap*) in 15 children with shared *ama1* alleles detected by single amplicon sequencing between the end of the dry season (May) and during their first clinical malaria case in the ensuing wet season (MAL). All boxplots indicate median ± IQR with all individual values plotted.

Appendix figure S2

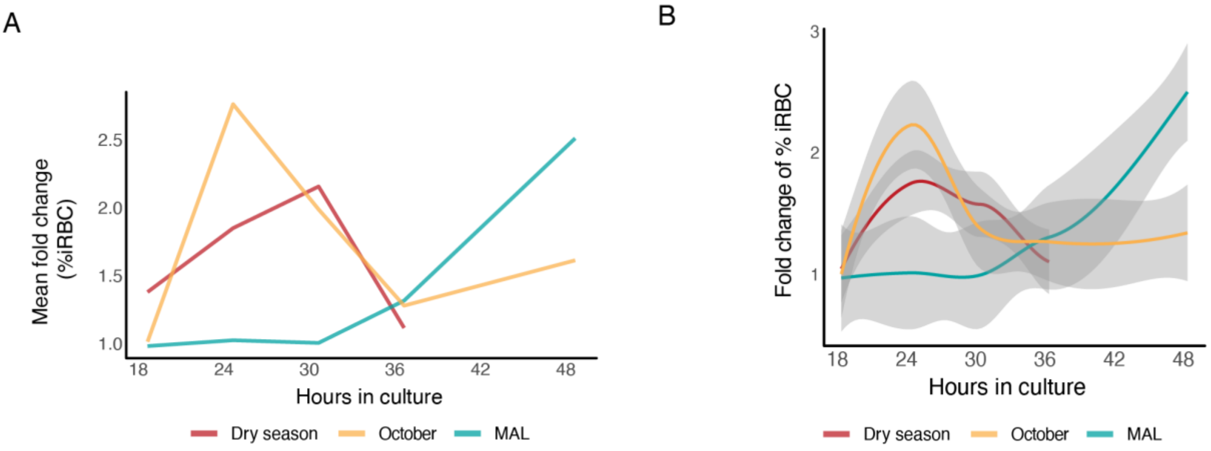

A. Mean fold change of percentage of infected red blood cells over equally spaced time intervals for the three sample groups; dry season (red, n=108), wet season asymptomatic in October (yellow, n=67) and malaria cases (aqua, n=39). **B.** Locally estimated scatterplot smoothing (Loess) model-based smooth curve fitting of fold change of % iRBCs over time using equally spaced time intervals. Dry season (red, n=108), wet season asymptomatic in October (yellow, n=67) and malaria cases (aqua, n=39). Lines correspond to the Loess-fitted curve whereas the gray-shaded areas represent the 95% confidence interval for the fitted values.

Appendix figure S3

A

B

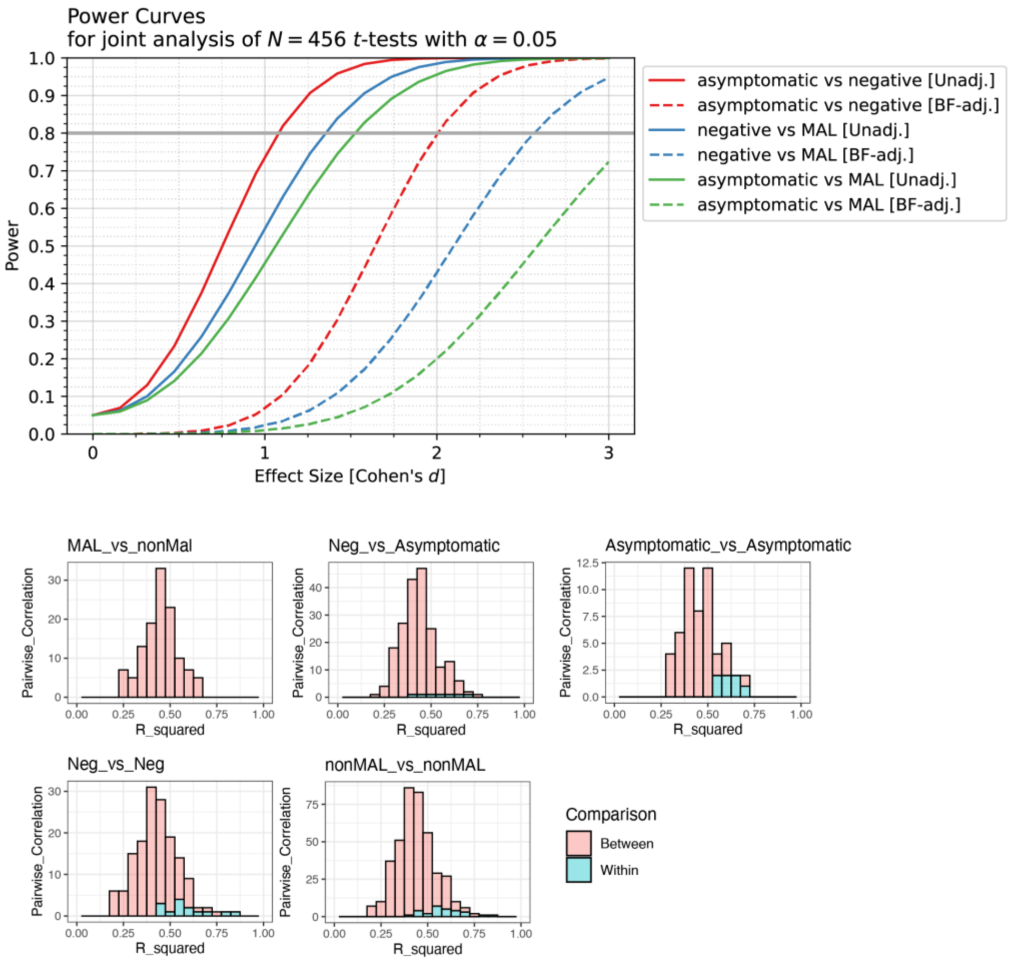

A. Power analysis using the sample sizes for each of the four experimental groups (asymptomatic, n=11; negative, n=21; dry-season, n=32; October, n=11; MAL, n=6) and the two-sided t-test used to detect statistically significant differences in metabolite abundance. Conservative, lower-bound on the power (y-axis) using Bonferroni-adjusted t-test is shown (broken line) or unadjusted (continuous line) for the different sample comparisons are represented with the Cohen's d-statistic (x-axis). **B.** Distribution of R-squared (R^2) value of a linear regression model of all possible pairwise combinations of metabolite abundance for all timepoints and individuals split by pairwise comparisons within the same individual (applies only to individuals with repeated measurements) and all timepoints between individuals. Individuals with no paired timepoints were excluded and since only the first malaria episode per person was included, the within comparison of MAL does not apply.

Appendix figure S4

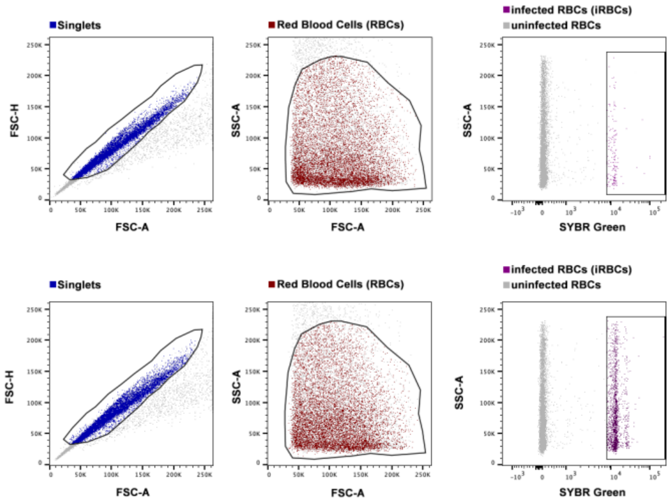

Gating strategy used to define infected RBCs of Malian samples at 0h and following in vitro culture. Top panel shows and example of low parasitaemia (n=179 iRBCs) and bottom shows an example of high parasitaemia (n=1783 iRBCs).
