# Supplementary material for: Infection length and host environment influence on Plasmodium falciparum dry season reservoir
Source: EMBO Mol Med. 2024 Sep 16;16(10):2349–75. doi: 10.1038/s44321-024-00127-w (PMC11473648; doi:10.1038/s44321-024-00127-w)
Supplement: Supplementary file 12 — Source data Fig. 3 [file 44321_2024_127_MOESM12_ESM.zip › Figure 3/3C-D/readme_3C-D.rtf]

Source data 3C-DN/A: not applicable Supplementation_type: Plasma supplementation per sample. May or October (Oct) plasma; or complete rpmi (RPMIc).Fold change calculated as %iRBC t(n)/%iRBC t(n−). Collection_Month: Month in which each sample was collected and grown in vitro. 
