# Supplementary material for: Infection length and host environment influence on Plasmodium falciparum dry season reservoir
Source: EMBO Mol Med. 2024 Sep 16;16(10):2349–75. doi: 10.1038/s44321-024-00127-w (PMC11473648; doi:10.1038/s44321-024-00127-w)
Supplement: Supplementary file 13 — Source data Fig. 4 [file 44321_2024_127_MOESM13_ESM.zip › Figure 4/4C/readme_4C.rtf]

Source data 4CClinical malaria dates of 64 children.ama1 haplotype data is present in supplementary table 6
