# Supplementary material for: Infection length and host environment influence on Plasmodium falciparum dry season reservoir
Source: EMBO Mol Med. 2024 Sep 16;16(10):2349–75. doi: 10.1038/s44321-024-00127-w (PMC11473648; doi:10.1038/s44321-024-00127-w)
Supplement: Supplementary file 14 — Source data Fig. 5 [file 44321_2024_127_MOESM14_ESM.zip › Figure 5/5E-G/Readme5F.rtf]

Source data 5FHighest increase plotted per subject: Timepoint to which the highest fold change was reported per sample ID. Timepoint: Timepoint of highest fold change, per subject. Fold change calculated as %iRBC t(n)/%iRBC t(n−). Month: Sample collection monthStudy: Andrade et al, reported in our previous study https://doi.org/10.1038/s41591-020-1084-0Study: New, reported in the current study 
