# Supplementary material for: Infection length and host environment influence on Plasmodium falciparum dry season reservoir
Source: EMBO Mol Med. 2024 Sep 16;16(10):2349–75. doi: 10.1038/s44321-024-00127-w (PMC11473648; doi:10.1038/s44321-024-00127-w)
Supplement: Supplementary file 14 — Source data Fig. 5 [file 44321_2024_127_MOESM14_ESM.zip › Figure 5/5E-G/Readme_5G.rtf]

Source Data 5GID: Sample IDiRBC_0h: Parasitemia measured by sample at 0h.Month: Sample collection monthPercRings: Percentage of rings measured at 0h Sample_type: Which category each sample corresponds to. 
