# Supplementary figures and images for: Infection length and host environment influence on Plasmodium falciparum dry season reservoir
Source: EMBO Mol Med. 2024 Sep 16;16(10):2349–75. doi: 10.1038/s44321-024-00127-w (PMC11473648; doi:10.1038/s44321-024-00127-w)

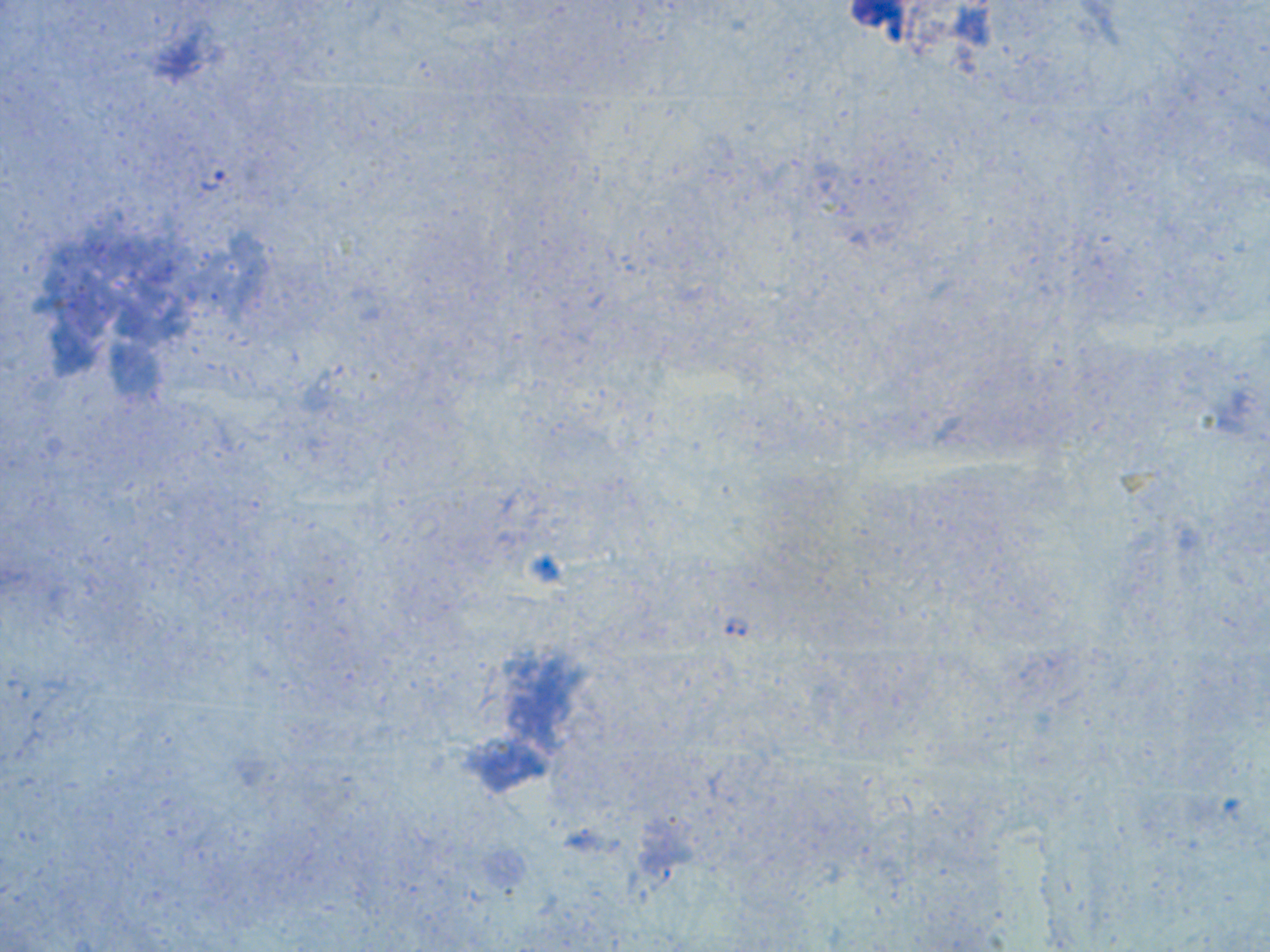

Supplement: Supplementary file 14 — Source data Fig. 5 [file 44321_2024_127_MOESM14_ESM.zip › Figure 5/5H/Oct19_K0812.19bmp.tif]

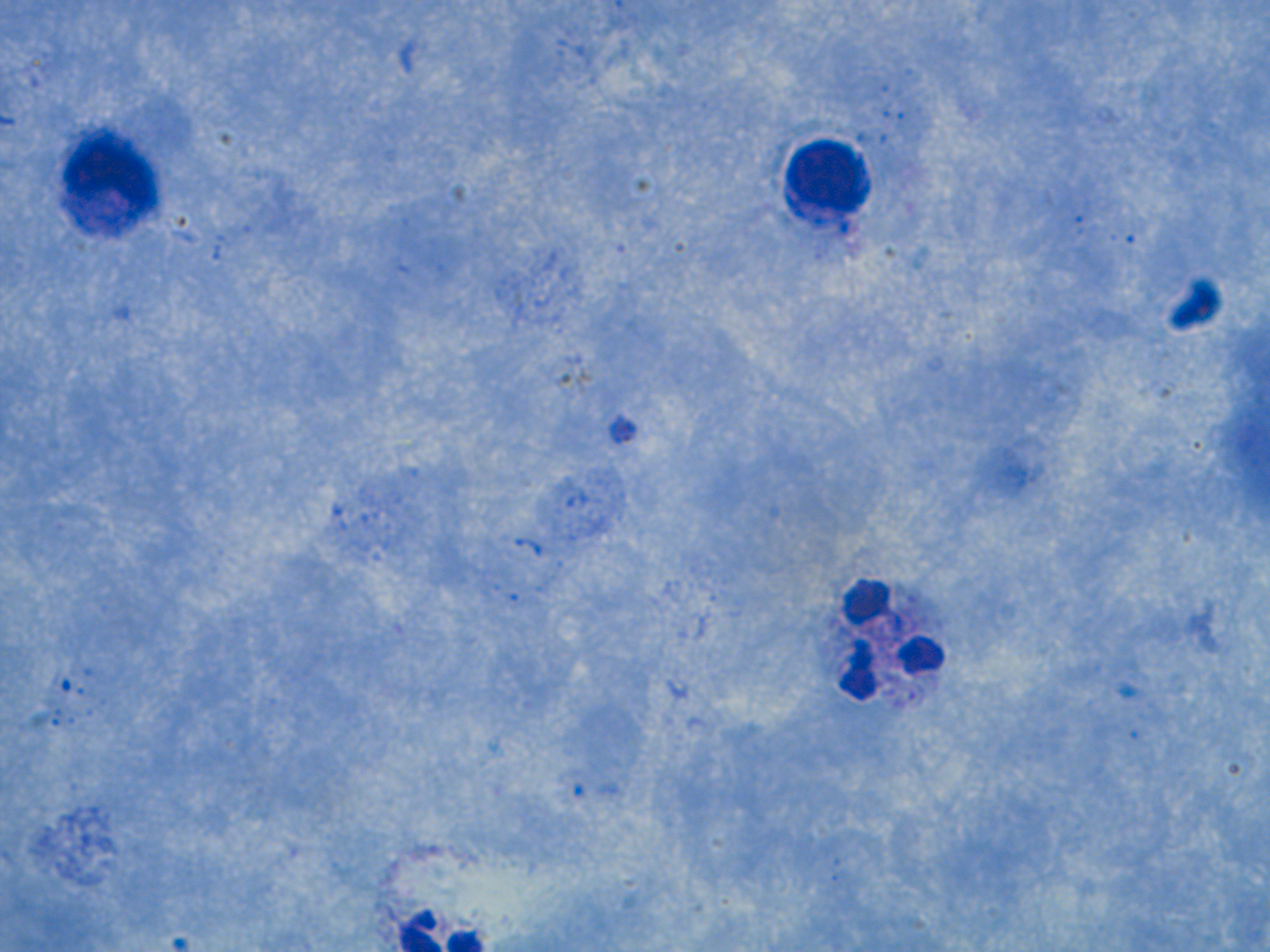

Supplement: Supplementary file 14 — Source data Fig. 5 [file 44321_2024_127_MOESM14_ESM.zip › Figure 5/5H/Oct19_K1053.7bmp.tif]

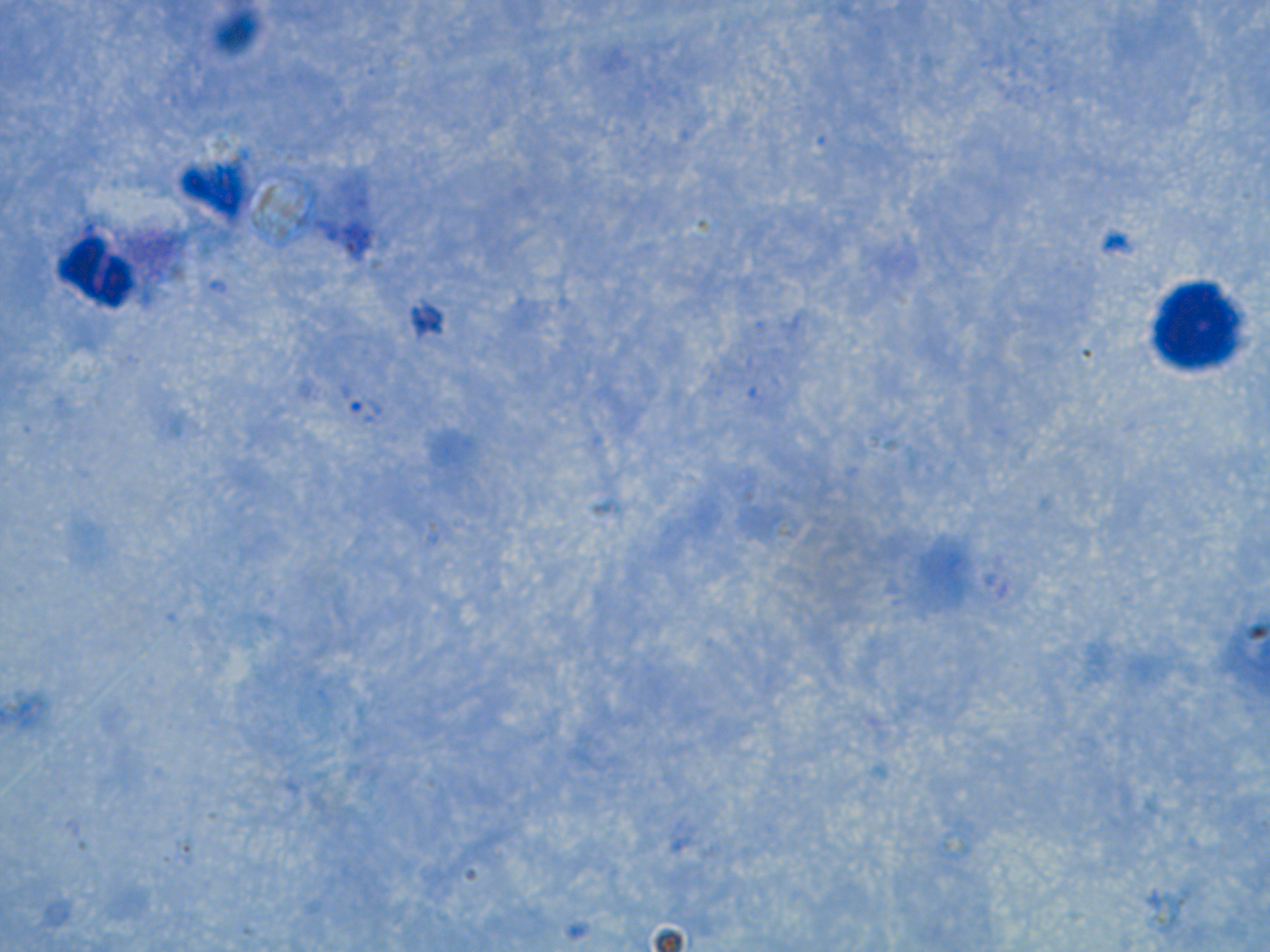

Supplement: Supplementary file 14 — Source data Fig. 5 [file 44321_2024_127_MOESM14_ESM.zip › Figure 5/5H/Oct19_K0632.tif]

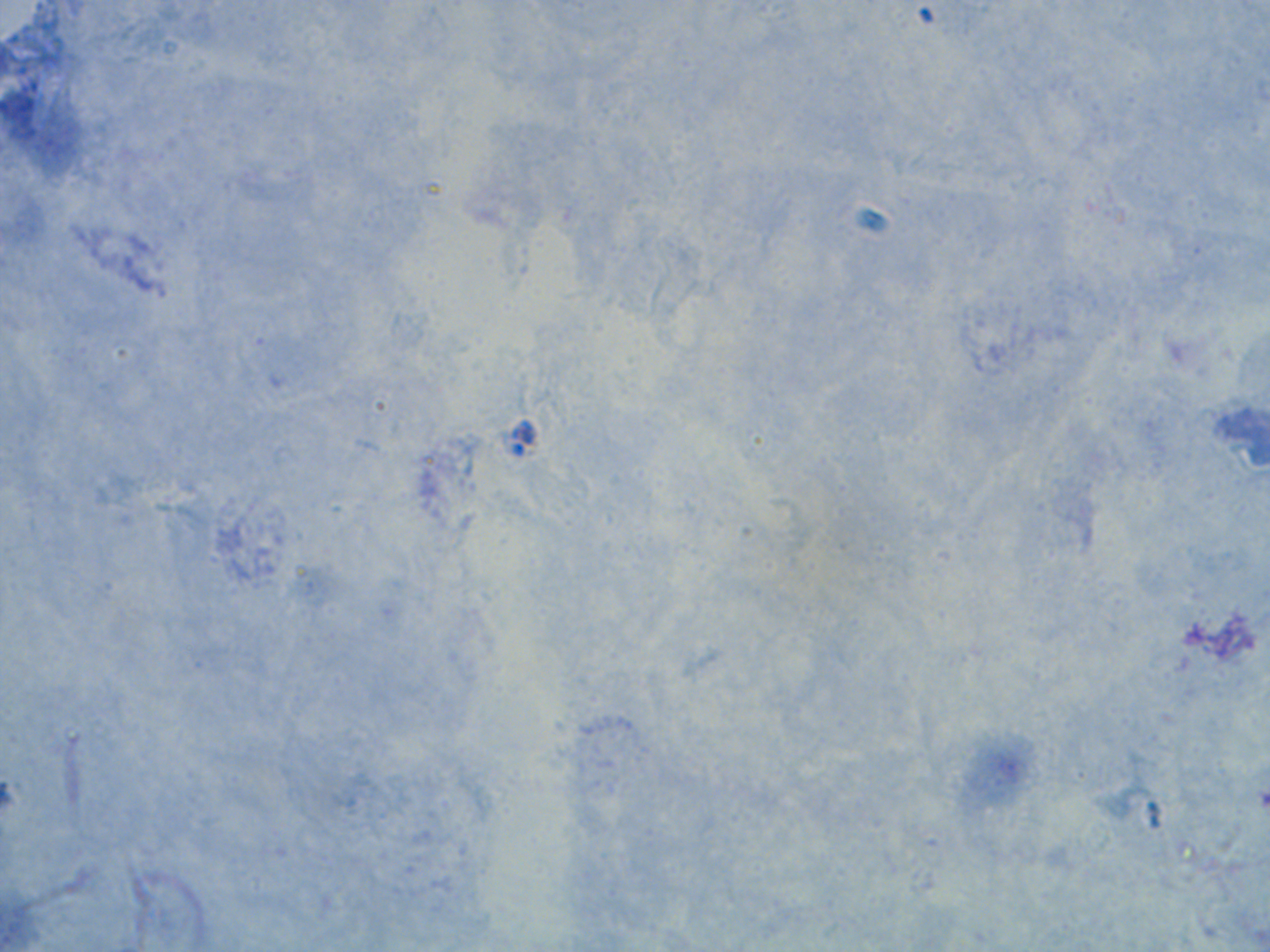

Supplement: Supplementary file 14 — Source data Fig. 5 [file 44321_2024_127_MOESM14_ESM.zip › Figure 5/5H/Oct19_K0812.14bmp.tif]

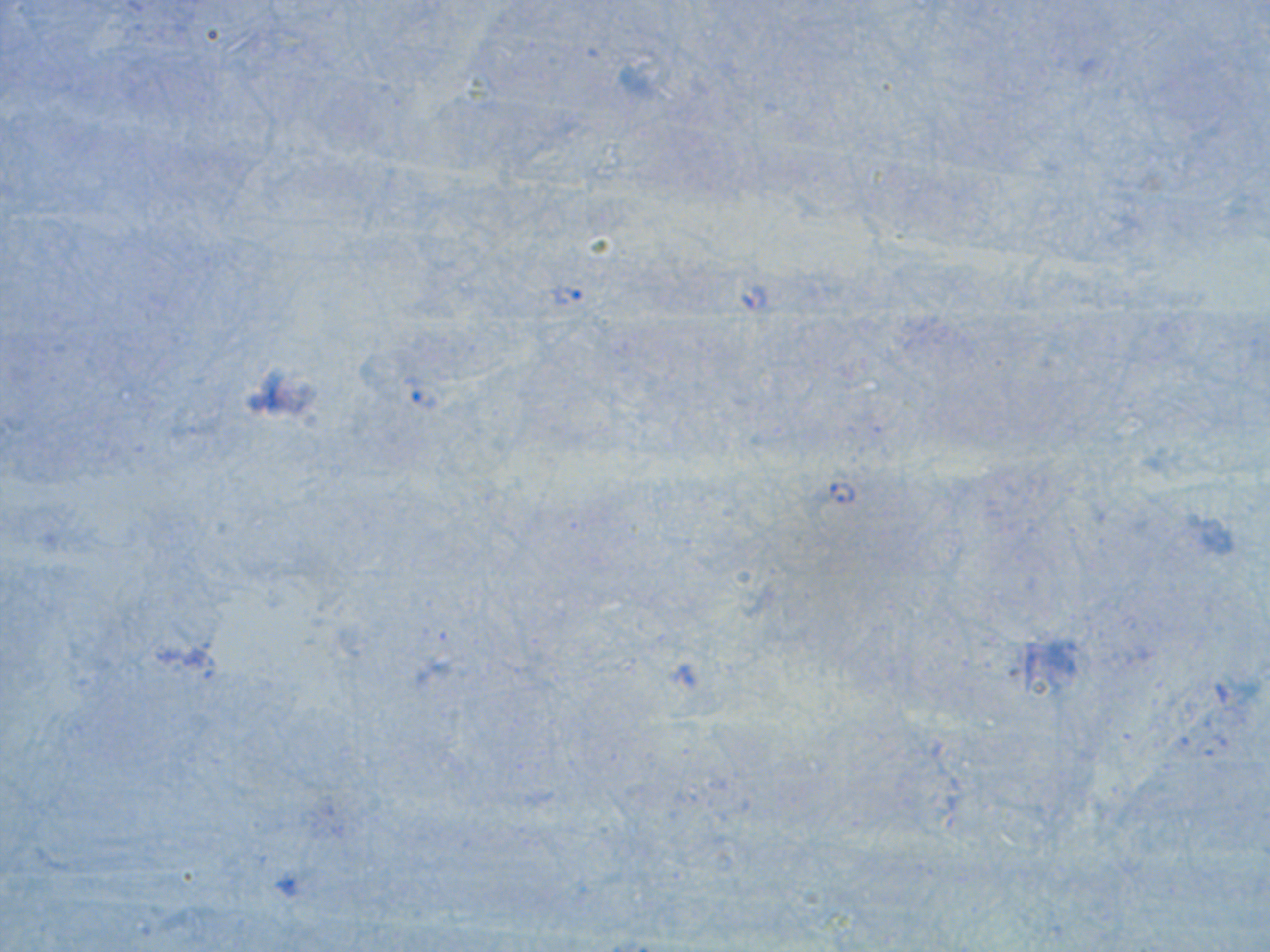

Supplement: Supplementary file 14 — Source data Fig. 5 [file 44321_2024_127_MOESM14_ESM.zip › Figure 5/5H/Oct19_K0573.12bmp.tif]

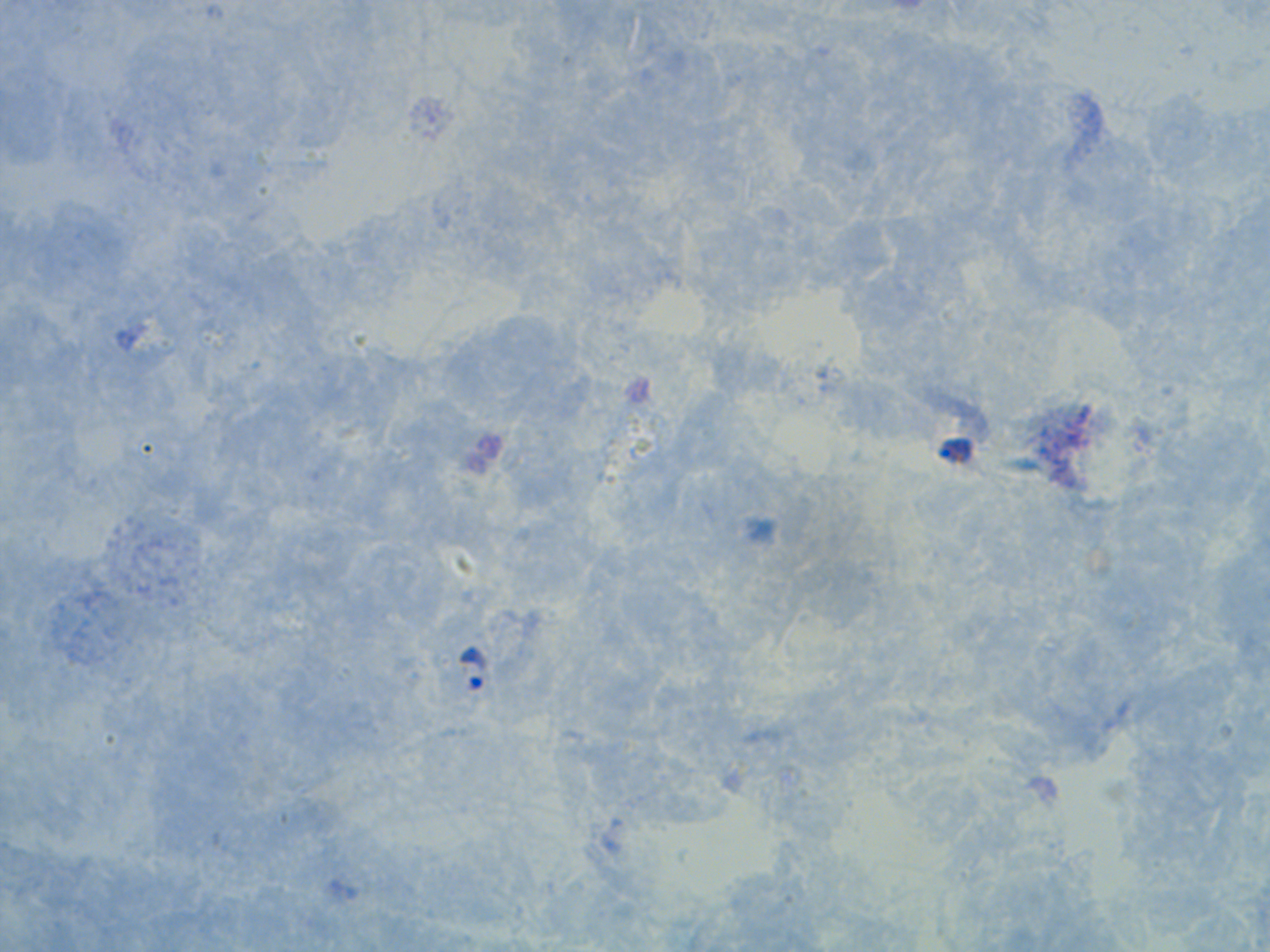

Supplement: Supplementary file 14 — Source data Fig. 5 [file 44321_2024_127_MOESM14_ESM.zip › Figure 5/5H/Oct19_K0084.5bmp.tif]

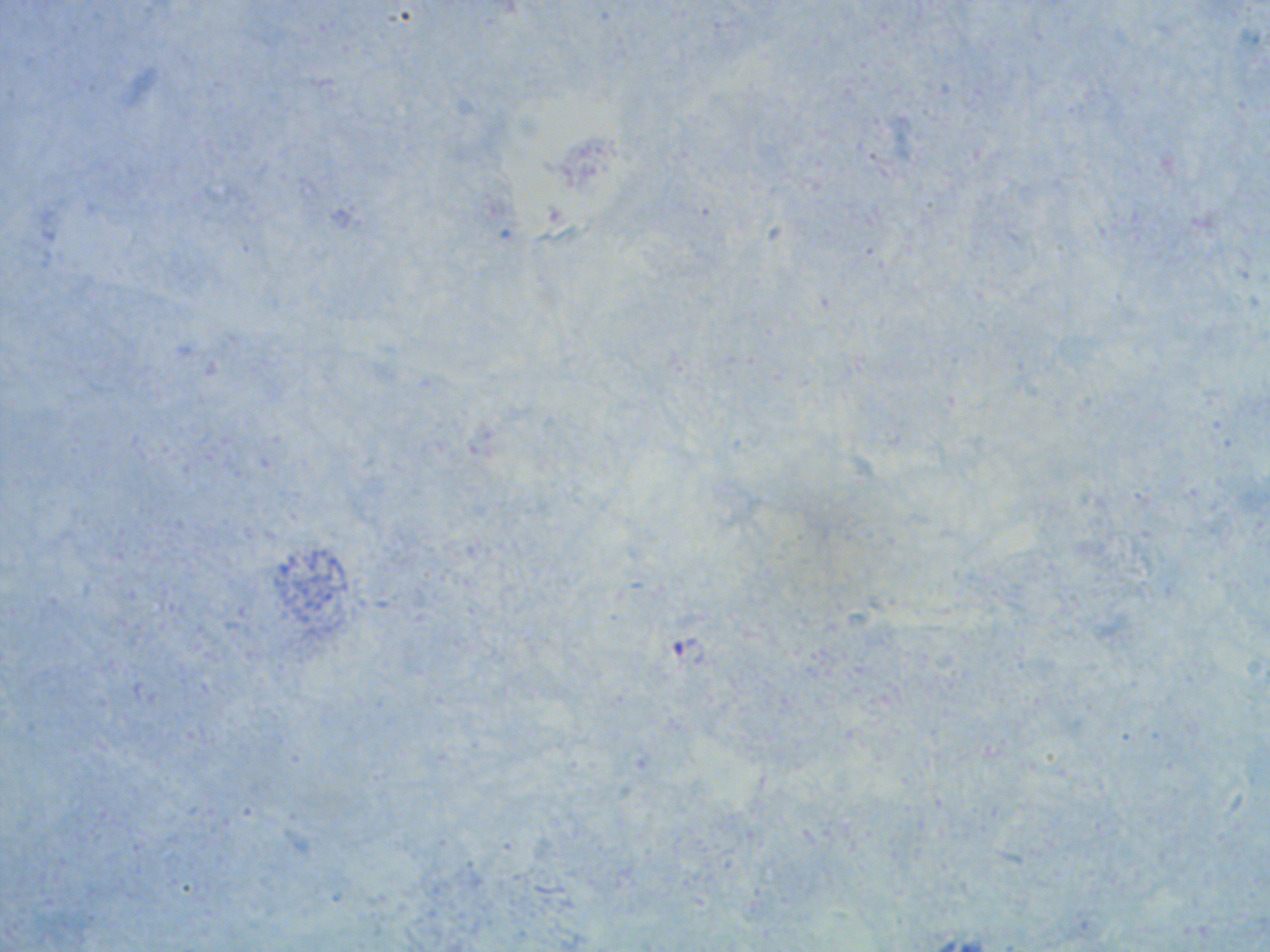

Supplement: Supplementary file 14 — Source data Fig. 5 [file 44321_2024_127_MOESM14_ESM.zip › Figure 5/5H/Oct19_K0999.4bmp.tif]

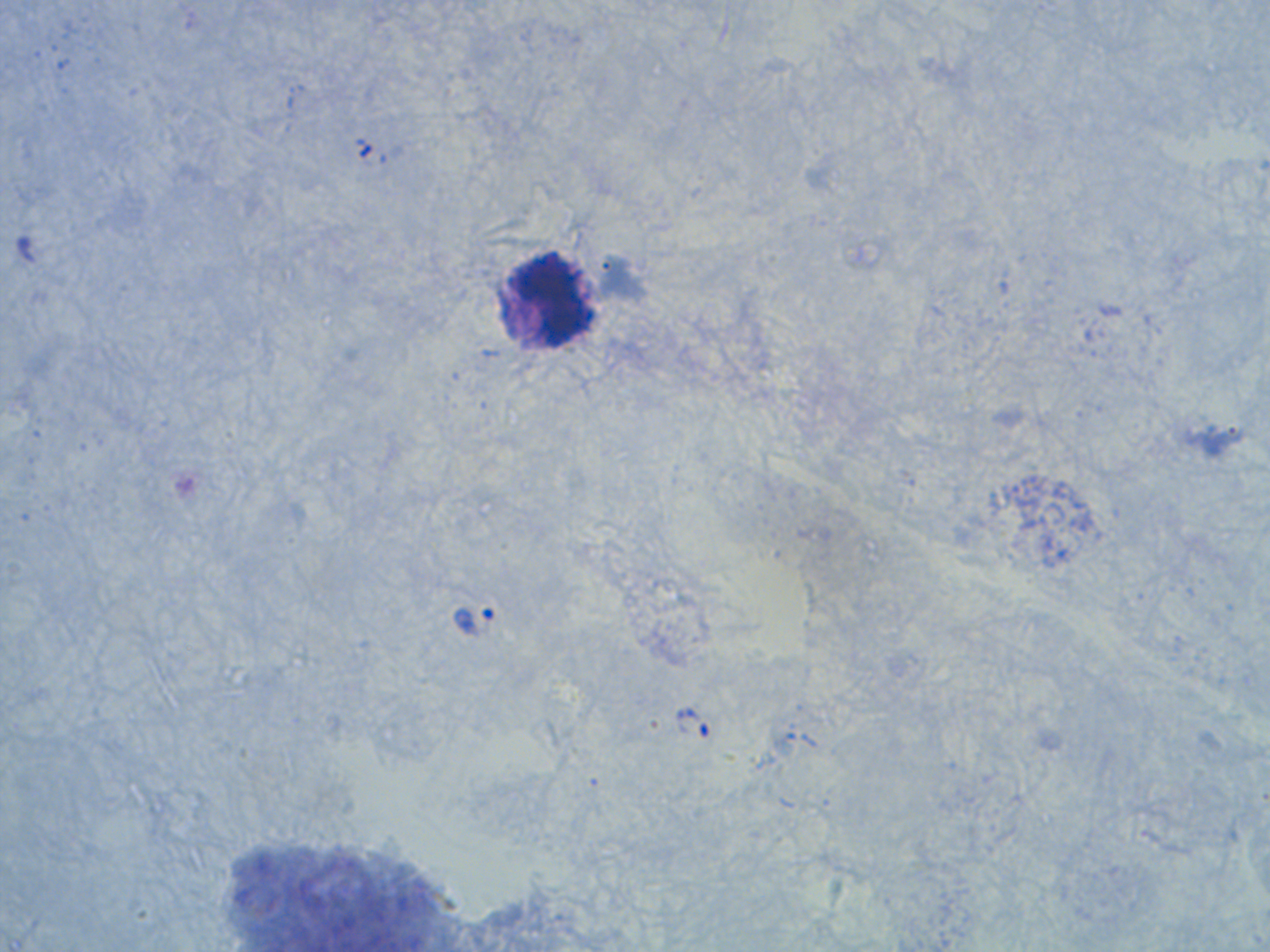

Supplement: Supplementary file 14 — Source data Fig. 5 [file 44321_2024_127_MOESM14_ESM.zip › Figure 5/5H/Oct19_K1020.19bmp.tif]
