# Supplementary material for: Infection length and host environment influence on Plasmodium falciparum dry season reservoir
Source: EMBO Mol Med. 2024 Sep 16;16(10):2349–75. doi: 10.1038/s44321-024-00127-w (PMC11473648; doi:10.1038/s44321-024-00127-w)
Supplement: Supplementary file 15 — Expanded View Figures [file 44321_2024_127_MOESM15_ESM.pdf]

Expanded View Figures

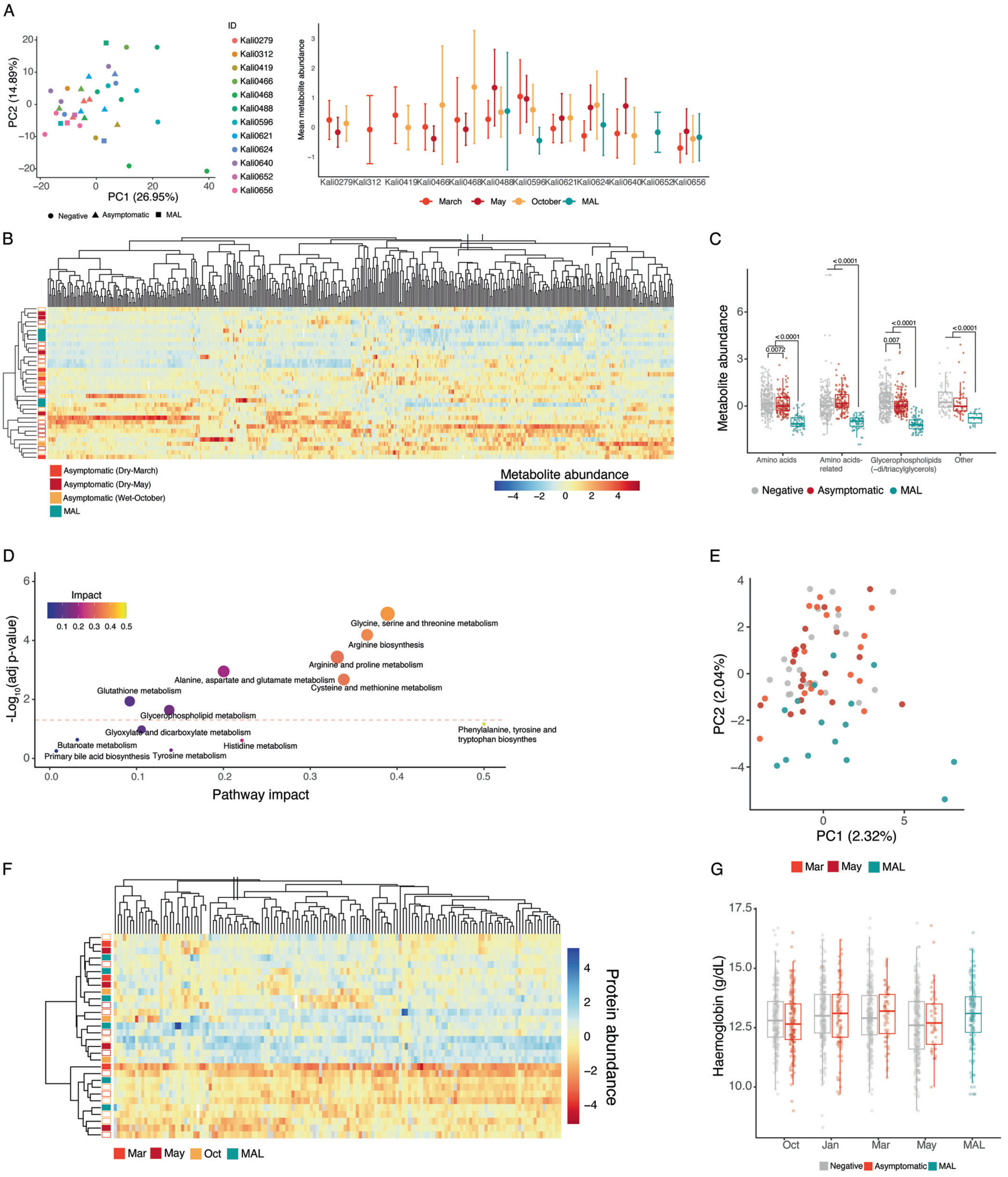

 © The Author(s)

Figure EV1. Plasma metabolites, lipids and proteins across seasons and clinical presentations.

(**A**) Principal component analysis (PCA) of 456 metabolites in the plasma of uninfected (Negative shown in circles) individuals ($n = 19$), obtained in March ($n = 7$), May ($n = 5$) and October ($n = 7$); *P. falciparum* asymptomatic carriers (Asymptomatic shown in triangles) ($n = 11$), obtained in March ($n = 4$), May ($n = 4$) and October ($n = 3$); and clinical cases during the wet season (MAL shown in squares) ($n = 5$) presenting the variance explained by the two first principal components between individuals based on their metabolite profiles and coloured by individual (left panel). Mean and standard deviation of the normalised metabolite abundance for each individual ($n = 11$ individuals and 35 samples) at the different timepoints included in the analysis (right panel). (**B**) A heatmap with hierarchical clustering showing the normalised and log2-transformed abundance of 456 metabolites (columns) of 35 plasma samples (rows) collected along the year, infection and clinical presentation status. Metabolites are listed in Dataset EV1. (**C**) Significantly different metabolites across all sample comparisons ($n = 39$) grouped by metabolite class and infectious status, including uninfected individuals (Negative), *P. falciparum* asymptomatic carriers (Asymptomatic) and clinical malaria cases (MAL). Boxplots indicate median ± IQR with all individual values plotted, pairwise Student's *t*-test and adjusted *p* values are shown. (**D**) Pathway enrichment analysis of the significant metabolites between clinical cases during the wet season and uninfected and *P. falciparum* asymptomatic carriers. Circles represent pathways with the colour scale and size adjusted to the pathway impact (x-axis) and number of hit metabolites, respectively. Statistical significance was determined with hypergeometric test for overrepresentation analysis, and impact calculated based on the relative centrality from pathway topology analysis with multiple testing correction. Significant pathways have an adjusted $p > 1.3$ (y-axis) and pathway impact >0 and are listed in Dataset EV3. (**E**) Principal component analysis (PCA) was performed on data from 73 samples and 20 lipid species, showing the variance explained by the two first principal components between individuals based on their lipid profiles. (**F**) A heatmap with hierarchical clustering showing protein abundance for each of the 146 proteins (columns) across 30 plasma samples (rows) collected along the year, infection and clinical presentation status. Proteins are listed in Dataset EV4.
(**G**) Haemoglobin levels in the blood of study participants above 5 years old collected along the year, infection and clinical presentation status ($n = 391$ study participants). Boxplots indicate median ± IQR with all individual values plotted.

© The Author(s)

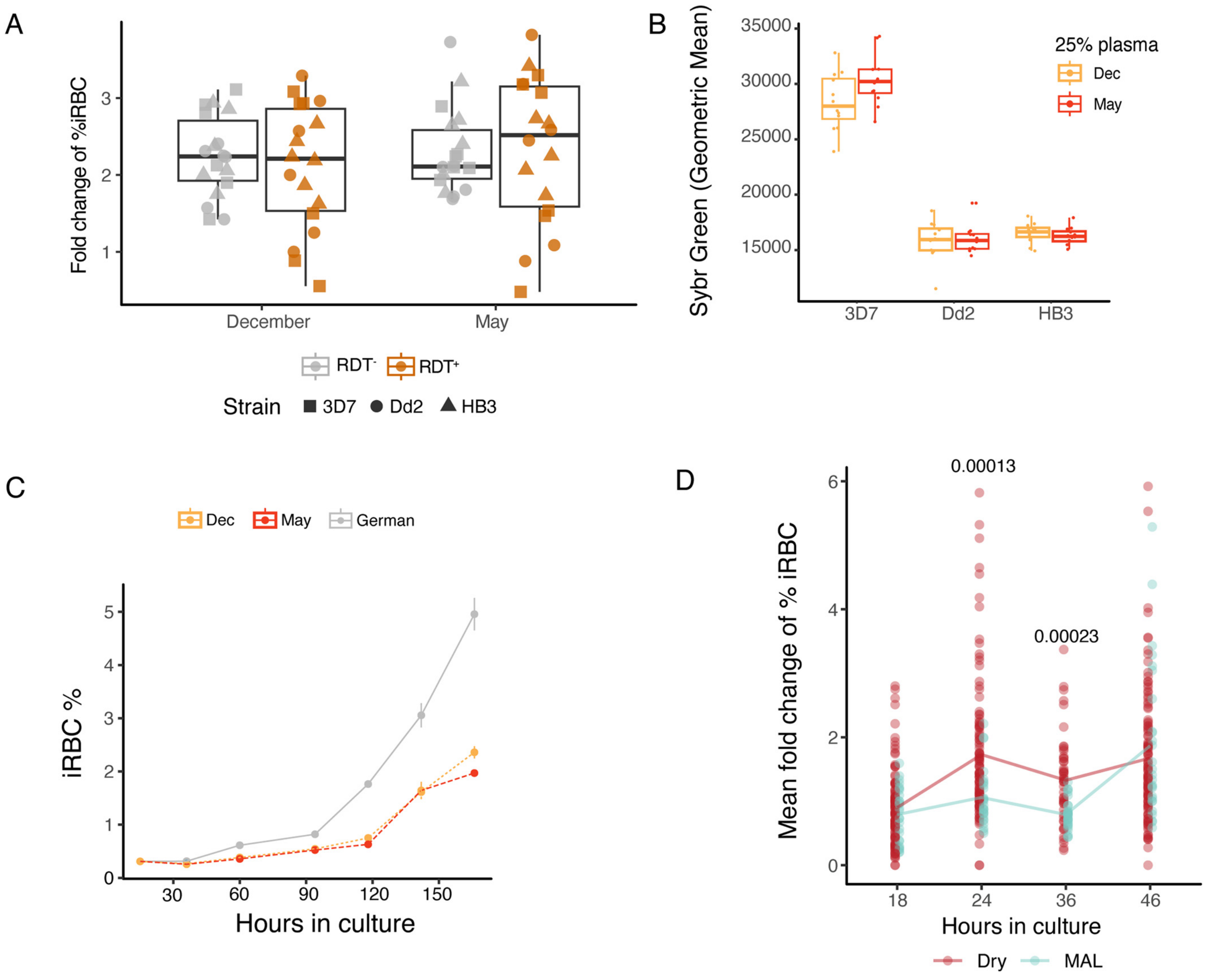

Figure EV2. Plasma from the dry and wet seasons have a similar effect on *P. falciparum* lab-adapted strains in vitro.

(**A**) Parasitaemia fold change of lab-adapted 3D7, Dd2 and HB3 *P. falciparum* strains after 60 h in vitro culture supplemented with 25% plasma from Malian donors, split by infection status by rapid diagnostic test of plasma donors (RDT$^-$, $n = 6$, RDT$^+$, $n = 6$). (**B**) Parasite development is measured as Sybr Green geometric mean in different *P. falciparum* lab-adapted strains cultured in vitro supplemented with December or May plasma from Malian donors ($n = 12$), measured at 36 h in culture (3D7) or 30 h in culture (Dd2 and HB3). (**C**) *P. falciparum* 2004 strain growth for over three 48 h cycles in vitro supplemented with 25% plasmas pooled from 12 Malian children in the beginning (December, $n = 3$) or end (May, $n = 3$) of the dry season, or from four German adults ($n = 3$). (**D**) Average fold change of iRBCs at 18, 24, 36 and 46 h after culture and across all plasma conditions for parasites obtained from the dry season ($n = 37$) or from clinical malaria cases ($n = 9$). Kruskal–Wallis and Wilcoxon pairwise test. Fold changes are defined as %iRBC t(n)/%iRBC t($n - 1$). All boxplots indicate median ± IQR with all individual values plotted.

 © The Author(s)

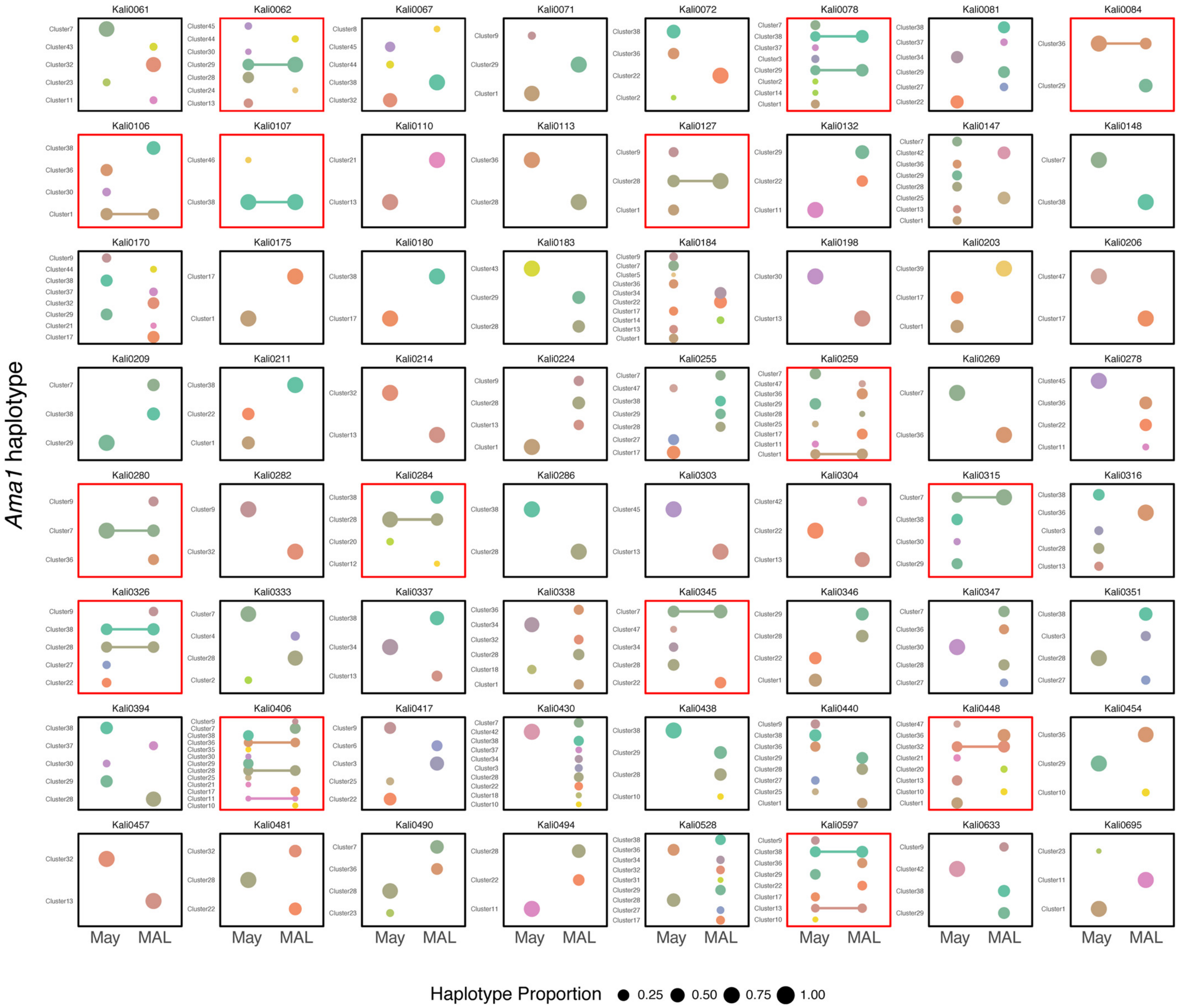

Figure EV3. Clinical malaria episodes are mainly caused by newly transmitted *P. falciparum* parasites.

ama1 haplotypes in paired samples from 64 children collected at the end of the dry season (May) and during their first clinical malaria case in the ensuing wet season (MAL). Red squares highlight children with shared haplotypes in May and MAL. Circle size represents haplotype proportion within each timepoint.

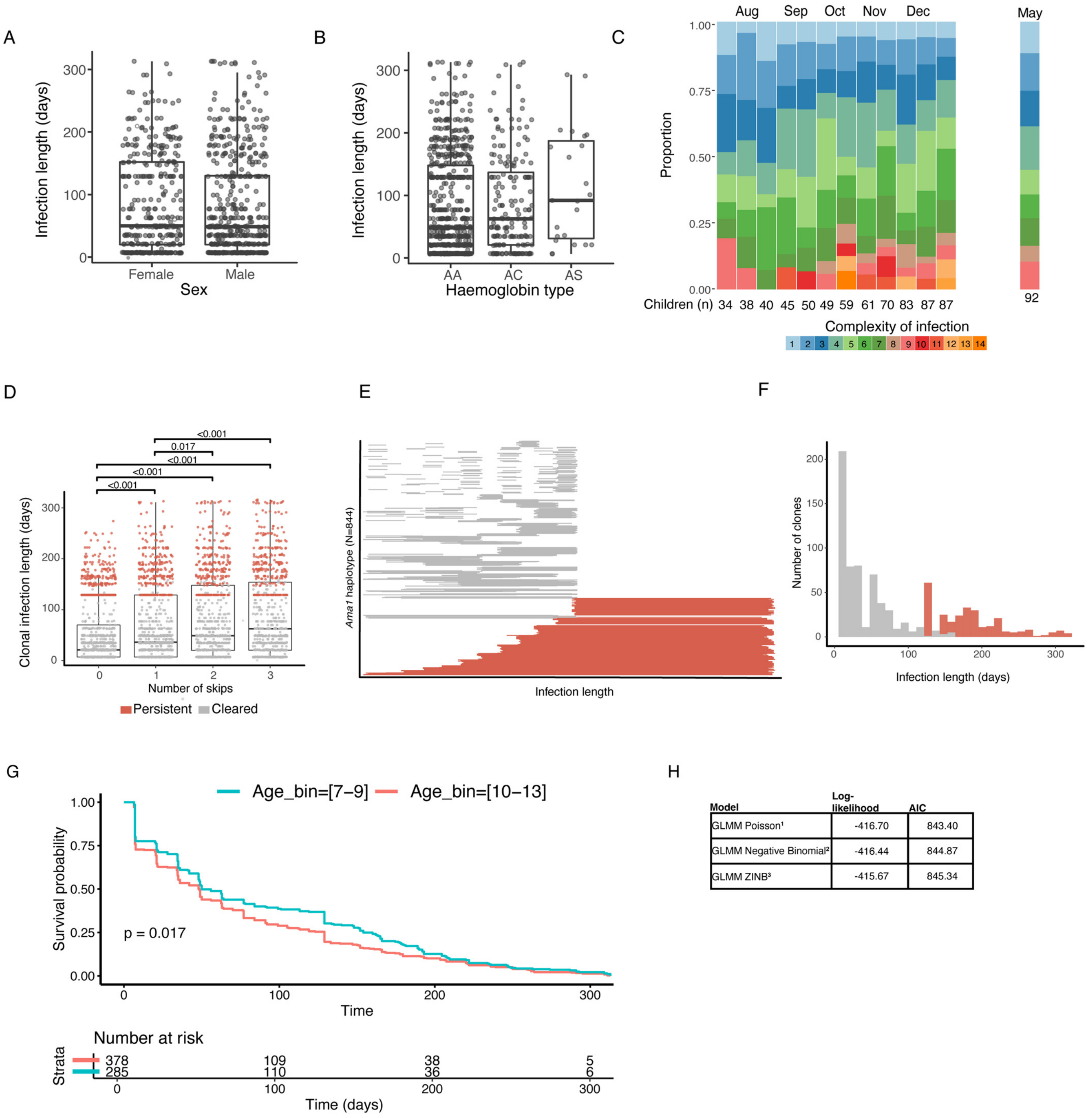

Model	Log-likelihood	AIC
GLMM Poisson[1]	-416.70	843.40
GLMM Negative Binomial[2]	-416.44	844.87
GLMM ZINB[3]	-415.67	845.34

Figure EV4. Determinants of infection length and complexity of infection in dry-season persisting infections.

Infection length of all *P. falciparum* clones (allowing for two skips) found in 92 Malian children separated by (**A**). Sex and (**B**). haemoglobin type; (AA $n = 71$, AC $n = 12$, AS $n = 9$). Boxplots indicate median ± IQR with all values plotted corresponding to the individuals and timepoints. (**C**) Complexity of infection (COI) of 92 asymptomatic children found PCR In May 2013 at the end of the dry season, analysed retrospectively over the 12 timepoints in the preceding wet season from December 2012 until July 2012, and at the end of the preceding dry season (May 2012). (**D**) Infection length of *P. falciparum* clones allowing 0, 1, 2 or 3 skips (negative timepoints of a particular *ama*1 clone within a series of positive ones). Brick dots show persistent infections, and grey dots represent clones cleared before the end of the dry season (ANOVA and Tukey multiple comparison test). Boxplots indicate median ± IQR. (**E**) Haplotypes are ordered by clonal infection length. (**F**) Distribution of clones and their infection length. Brick lines show persistent infections, and grey lines represent clones cleared before the end of the dry season. (**G**) Kaplan–Meier analysis showing the probability of survival of *P. falciparum* clones (y-axis) over time, from the start of the wet season (July 2012) to the end of the dry season (May 2013) (x-axis) in individuals of 7–9 (aqua) and 10–13 (coral) years old, with the log-rank test used to determine statistical differences. (**H**) Summary of three generalised mixed-effect models used showing the Akaike Information Criteria and the log-likelihood for the selected model, GLMM Poisson (1), GLMM negative binomial model (2) and GLMM ZINB (3).

© The Author(s)

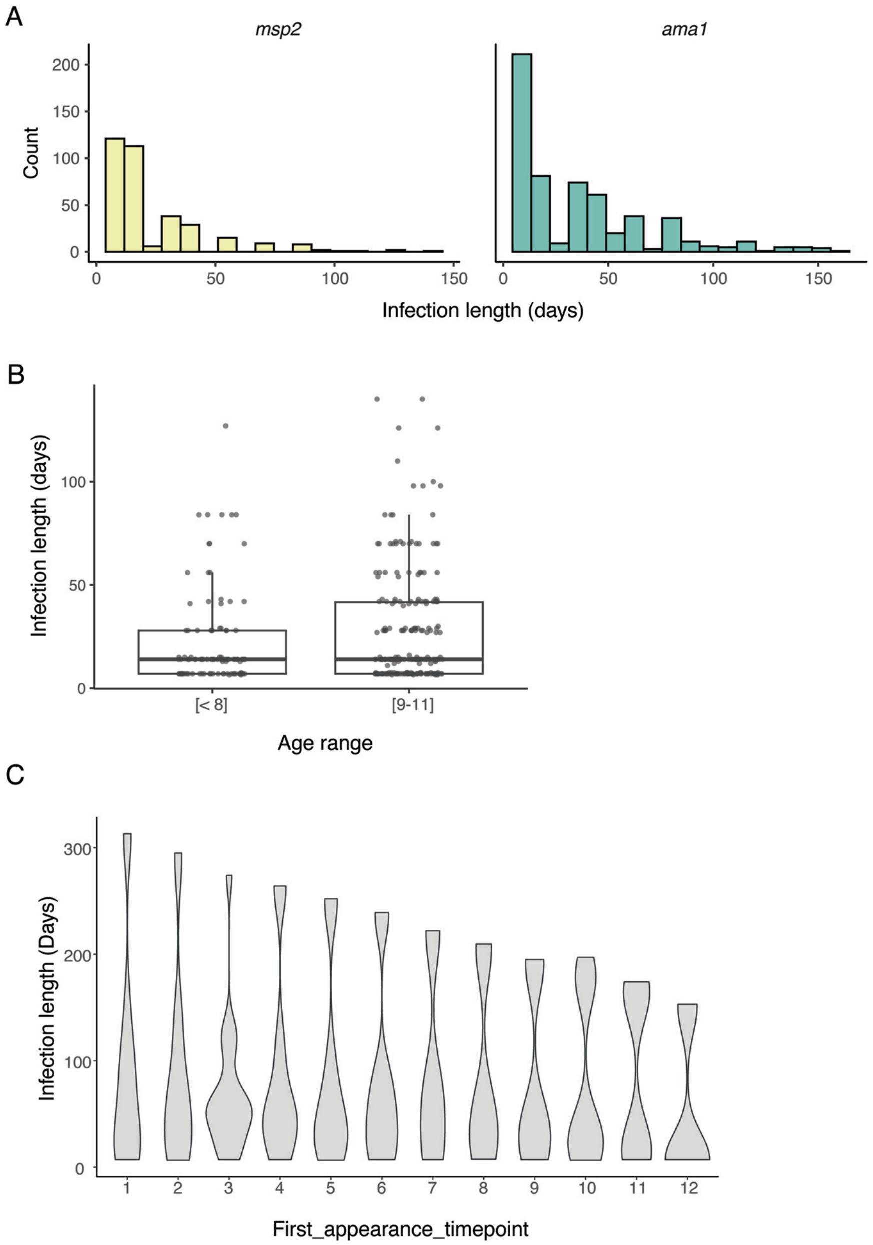

Figure EV5. Distribution of infection length of *P. falciparum* clones using two genotyping approaches.

(**A**) Distribution of infection length in days of *P. falciparum* clones detected by *msp2* genotyping (yellow) and *ama1* genotyping methods (green), showing length with two skips of 346 and 582 clonal infections, respectively (x-axis). The Y-axis corresponds to the number of clones per genotyping method. (**B**) Infection length determined by *msp2* genotyping of younger (5–8) or older (9–11) children (**C**) Infection length distribution using *ama1* amplicon sequencing and 2 skips of individual clones across all individuals for the 12 timepoints spanning the transmission season (July to December, 2012).

© The Author(s)
